# Iodide Salt Surface Etching Reduces Energy Loss in CdTe Nanocrystal Solar Cells

**DOI:** 10.3390/nano15151180

**Published:** 2025-07-31

**Authors:** Jielin Huang, Xuyang Wang, Yilin Chen, Zhenyu Chen, Qiaochu Lin, Qichuan Huang, Donghuan Qin

**Affiliations:** 1 School of Materials Science and Engineering, South China University of Technology, Guangzhou 510640, China; 202230271209@mail.scut.edu.cn (J.H.); 202230274026@mail.scut.edu.cn (X.W.); msylchen@mail.scut.edu.cn (Y.C.); 202230270257@mail.scut.edu.cn (Z.C.); 202130273624@mail.scut.edu.cn (Q.L.); hqcscut@outlook.com (Q.H.); 2 State Key Laboratory of Luminescent Materials & Devices, Institute of Polymer Optoelectronic Materials & Devices, South China University of Technology, Guangzhou 510640, China

**Keywords:** CdTe, solar cells, etching, nanocrystalline

## Abstract

CdTe nanocrystals (NCs) have emerged as a promising active layer for efficient thin-film solar cells due to their outstanding optical properties and simple processing techniques. However, the low hole concentration and high resistance in the CdTe NC active layer lead to high carrier recombination in the back contact. Herein, we developed a novel 2-iodothiophene as a wet etching solution to treat the surface of CdTe NC. We found that surface treatment using 2-iodothiophene leads to reduced interface defects and improves carrier mobility simultaneously. The surface properties of CdTe NC thin films after iodide salt treatment are revealed through surface element analysis, space charge limited current (SCLC) studies, and energy level investigations. The CdTe NC solar cells with 2-iodothiophene treatment achieved power conversion efficiency (PCE) of 4.31% coupled with a higher voltage than in controlled devices (with NH_4_I-treated ones, 3.08% PCE).

## 1. Introduction

Solution-processed nanocrystal (NC) materials have garnered significant interest due to their potential for creating low-cost and high-performance optoelectronic devices. Among them, cadmium telluride (CdTe) NCs possess an ideal bandgap for single-junction devices, aligning well with the solar spectrum and ensuring high photon absorption efficiency [1,2,3,4,5,6]. Moreover, due to their solution processability, minimal raw material usage, and simple fabrication processes, these characteristics make CdTe NCs a promising candidate for high-efficiency photovoltaic applications [7,8,9]. However, CdTe NC solar cells currently face a significant challenge: the back contact issue. Due to low doping concentrations and the self-compensation effect, along with surface oxidation, severe recombination at the back contact occurs [10,11,12]. This limits the overall efficiency and further development of CdTe NC solar cells. To address this issue, the most commonly used methods are copper doping, introducing a hole transport layer between CdTe NC film and the contact electrode, or surface etching [13,14,15]. Among these methods, the introduction of a hole transport layer has achieved significant success in mediating charge extraction and boosting the overall performance of solar cells. To effectively reduce carrier recombination at the semiconductor–metal interface, selecting a hole transfer material (HTM) with a high work function is essential. For instance, by employing crosslinkable conjugated polymers like poly (diphenylsilane-co-4-vinyl-triphenylamine) and poly (phenylphosphine-co-4-vinyl-triphenylamine) [16,17], CdTe NC solar cells with a configuration of ITO (indium tin oxide)/ZnO/CdSe/CdTe/HTM/Au were fabricated, and over 9% power conversion efficiency (PCE) was achieved [16]. This improvement is due to the formation of a dipole layer between the CdTe NC and hole transport layer (HTL) interface. The dipole layer not only enhances the built-in electric field but also reduces the recombination of carriers at the back contact, thereby improving the collection efficiency and overall performance of the solar cell [14]. Other HTMs like MoO_x_, Spiro (2,2′,7,7′-tetrakis [N,N-di(4-methoxyphenyl)amino]-9,9′-spirobifluorene) are also applied in CdTe NC solar cells with a similar structure and exhibit improved performance [18,19]. Nevertheless, despite their promising potential, organic HTLs are still confronted with challenges associated with contact resistance, largely attributed to the intrinsically high resistance of organic materials. As a result, a portion of the current is hindered by the parasitic resistance within the organic HTL. Most highly efficient vacuum thermal evaporated CdTe thin-film solar cells employ a Cu/Au back contact to address this issue [20,21]. Immediately following the deposition of the CdTe thin-film, a thin layer of Cu (around 1 nm) is evaporated, enabling the formation of a Cu-doped CdTe layer. This layer functions as an HTL, enhancing the hole concentration and extending the carrier lifetime [22]. Despite the commercial success of the copper-doping technique, it has its drawbacks. Excessive Cu can diffuse into the CdTe active layer along grain boundaries [23,24,25]. In the case of solution-processed CdTe NC solar cells, the numerous crystal boundaries within the CdTe NC active layer pose a problem. Although the copper-doping technique has been effectively utilized in CdTe thin-film cells fabricated using vacuum processes, in solution-processed CdTe NC solar cells, the active layer needs to be polished or refined. This is because the copper-doping technique, which is successful in vacuum-processed CdTe thin-film cells, is destructive here [20].

To control the diffusion of Cu in the CdTe active layer, wet chemical etching is usually performed before the deposition of Cu/Au contact in the case of traditional CSS/vacuum-processed CdTe thin-film solar cells [26,27]. The primary etching solutions employed encompass nitric acid–phosphoric acid and bromine–methanol. Nevertheless, the aforementioned etching solutions are inapplicable to CdTe NC solar cells. The rationale behind this is that the NCs inherently possess numerous grain boundaries, which facilitate rapid diffusion of the etching solution along these boundaries. This, in turn, leads to severe corrosion of the thin film and ultimately its detachment. Our prior research has substantiated this phenomenon [14]. Consequently, we have engineered a novel chemical vapor etching technique to circumvent direct contact between the solution and the thin film, and initial successes have been realized. Herein, we introduce a novel surface etching process for the CdTe NC thin film that reduces surface oxides and minimizes interface recombination using 2-iodothiophene methanol solution. As the etching process is relatively mild, the competition between oxidation and the formation of element Te is carefully controlled by controlling the concentration. Through precise control of the surface etching process, we reconstructed the surface chemistry of CdTe NCs, achieving improved monodispersity and a significant reduction in surface oxides. This suppression of surface oxides, confirmed through X-ray photoelectron spectroscopy (XPS), leads to a lower density of trap states. As a result, enhanced carrier lifetimes and longer diffusion lengths were observed, contributing to the fabrication of CdTe NC solar cells with a power conversion efficiency (PCE) of 4.31%—representing an over 30% improvement compared to control devices etched using conventional NH_4_I (3.08%). This work provides new insights into the role of surface oxides in CdTe NC photovoltaics and introduces an effective surface engineering strategy to enhance device performance.

## 2. Experimental Procedure

**Figure 1:**
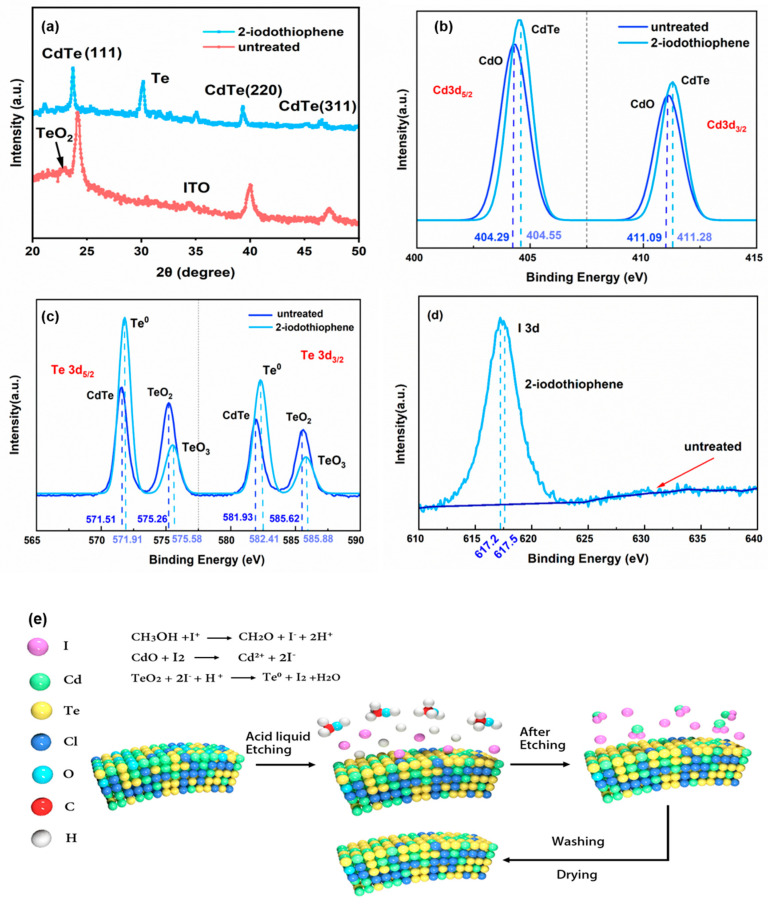
(**a**) The XRD pattern of the NC thin film. The XPS spectra of (**b**) Cd3d, (**c**) Te3d, and (**d**) I3d elements of the CdTe NC film with/without 2-iodothiophene treatment; (**e**) schematic diagram of the CdTe NC film after 2-iodothiophene treatment.

Zinc oxide (ZnO) precursors, CdSe, and CdTe NCs were synthesized in accordance with methodologies previously documented [15]. The ITO/ZnO/CdSe/CdTe structure, featuring a single layer of ZnO, two layers of CdSe NCs, and four layers of CdTe NCs, was prepared through a sequential solution-based layer-by-layer technique, as previously documented in reference [13]. The thickness of all of the active layers (ZnO/CdSe/CdTe) discussed in this paper was kept to ~300 nm (the transmittance of ITO/ZnO/CdSe/CdTe was about 50%, which permitted the fabrication of semi-transparent thin-film solar cells, as shown in Figure S1). The active area of the solar cells produced using this method measures 0.16 cm^2^. The fabrication process is illustrated in Figure 1e. The ITO/ZnO/CdSe/CdTe substrate was subjected to treatment with a 2-iodothiophene-methanol (or NH_4_I) solution at varying concentrations (from 5 μg/mL to 60 μg/mL), permitting a 20 s interval for the reaction between 2-iodothiophene and CdTe to achieve completion. Subsequently, the substrate underwent several rinses in methanol and was dried using a nitrogen gun. Ultimately, a gold (Au) electrode was deposited on the substrate to serve as the back-contact electrode through the process of vacuum thermal evaporation. The detailed device fabrication process can be found in our previous reports [28,29].

## 3. Results and Discussion

To evaluate the etching efficacy of iodized salt, we studied the X-ray diffraction (XRD) and photo-electron spectroscopy (XPS) results of untreated and 2-iodothiophene-treated CdTe NC films. The detailed crystal structures of the CdTe NCs with/without iodine salt treatment were further analyzed via XRD (Figure 1a). The XRD pattern of all samples exhibited diffraction peaks at 24.2°, 41.9°, and 47.6°, corresponding to the (111), (220), and (311) planes of the zinc-blende CdTe structure. Notably, in the samples treated with iodine salt, characteristic diffraction patterns with peaks at approximately 29.8° and 35.3° were observed, which correspond to the (101) and (102) planes of the wurtzite Te phase (similar results can be found in the case of the NH_4_I-treated sample). The XPS pattern of the CdTe NC thin film with/without 2-iodothiophene treatment (NH_4_I treatment produced similar results) is depicted in Figure 1b–d, with all spectra calibrated using the carbon C 1s core level as a reference [30] (binding energy at 284.8 eV). We observed a shift of the Cd 3d and Te 3d XPS peaks to a higher binding energy for the treated NC film compared with the peaks of the controls (Figure 1b–d and Table 1). For instance, the Cd 3d5/2 band depicted in Figure 1b for the control was resolved into two Gaussian bands with peak positions at 404.29 eV and 411.09 eV, attributed to Cd in CdO and CdTe, respectively [30]. In contrast, the Cd 3d5/2 band for the treated film, as shown in Figure 1b, was situated at 404.55 eV and 411.28 eV, corresponding to Te-Cd bonds in CdTe [30,31]. Similar results can be found in the case of the Te 3d5/2 band (Figure 1c). From Figure 1d, one can see that the I 3d band is located at 617 eV for treated samples, while no I signal is found in the control samples. Therefore, it is evident that the surface of the 2-iodothiophene-treated CdTe NC film comprised elemental tellurium, CdTe, I-, and cadmium oxide (CdO). Furthermore, upon conducting a surface elemental analysis of the NC thin film (refer to Table 1), it was observed that the Cd/Te ratio for the control samples was 1.58:1, whereas for the 2-iodothiophene-treated samples, the ratio was 1.45:1. This disparity distinctly indicates the formation of a Te-rich surface subsequent to 2-iodothiophene treatment.

Upon analyzing the XRD and XPS outcomes, a schematic reaction of CdTe NCs incorporating 2-iodothiophene is depicted in Figure 1e. The hydrolysis of 2-iodothiophene yields elemental iodine. Subsequently, an ion exchange reaction ensues, leading to the production of elemental tellurium (Te). Concurrently, the CdO that precipitates on the surface of the CdTe NC film is dissolved by the hydroiodic acid (HI) extracted during the methanol washing procedure, culminating in the creation of a TeO_2_ layer on the surface.

**Figure 2:**
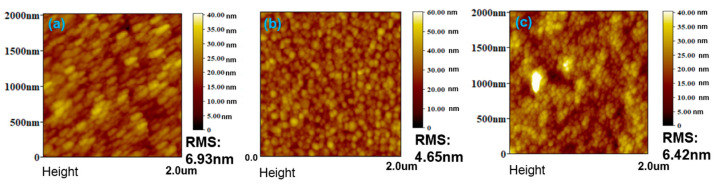
AFM images of ITO/ZnO/CdSe/CdTe (**a**) without etching, (**b**) with NH_4_I etching, and (**c**) with 2-iodothiophene etching.

To investigate the morphological changes of the CdTe thin films before and after treatment with 2-iodothiophene or NH_4_I methanol solutions of varying concentrations, atomic force microscopy (AFM) was employed to characterize the surface topography of CdTe/ITO substrates. The root-mean-square (RMS) roughness was obtained from a single representative AFM scan over a small area (2 × 2 μm^2^). As shown in Figure 2a, the pristine CdTe surface exhibits a relatively smooth morphology with an RMS roughness of 6.93 nm, indicating a compact and uniform film structure. After 2-iodothiophene or NH_4_I treatment, significant morphological changes were observed. Specifically, at a concentration of 5 µg/mL, a consistent decrease in RMS roughness was noted: 6.42 nm for 2-iodothiophene and 4.65 nm for NH_4_I. This trend suggests that after chemical etching, the CdTe reacts with 2-iodothiophene or NH_4_I and has a smoother surface, possibly due to the removal of surface irregularities or reorganization of CdTe NCs, which is beneficial for decreasing interface recombination and increasing carrier collection efficiency. However, it is important to note that an increase in the concentration of 2-iodothiophene methanol results in an increase in the RMS value, as shown in Figure S2. Conversely, the utilization of NH_4_I methanol as an etching solution results in the presence of significant pinholes throughout the films at a high concentration of 1 mg/mL, and the NC film has the potential to delaminate from the substrate when further increasing the concentration (Figure S3). A large RMS would increase the number of defect centers and increase the charge recombination in the interface of CdTe.

**Figure 3:**
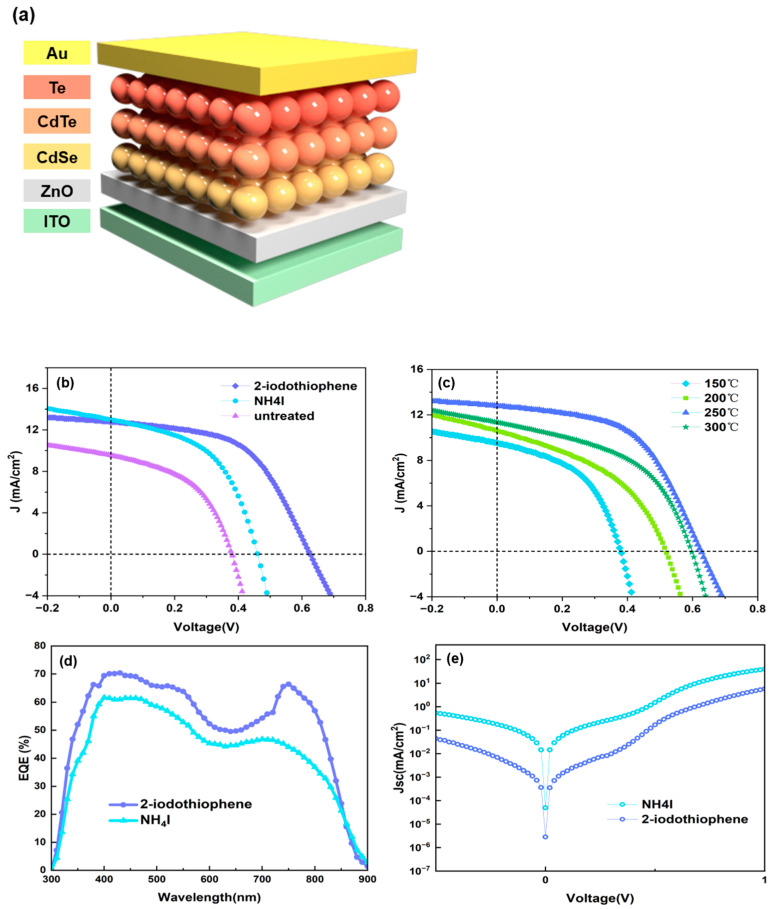
(**a**) Diagram of the cell. (**b**) *J-V* curves of CdTe NC devices subjected to different conditions under AM 1.5G (100 mW/cm^2^) illumination. (**c**) *J-V* curves of CdTe NC devices with 2-iodothiophene etching under different annealing temperatures. (**d**) EQE spectra and (**e**) dark *J-V* curves of devices with NH_4_I and 2-iodothiophene etching.

It has been established that a surface enriched with tellurium is advantageous for reducing carrier interface recombination and contact resistance. Previous research has demonstrated the efficacy of NH_4_I in the etching of CdTe thin-film surfaces [32]. Compared to etching solutions such as bromine–methanol or phosphoric acid–nitric acid mixtures, NH_4_I offers a more gentle and controllable etching process with comparable effectiveness. Nonetheless, due to the abundance of NC grain boundaries, molecules with high polarity have a propensity to diffuse along these boundaries, resulting in film delamination and device shunting—a phenomenon previously identified in our research [14]. In this study, we propose the use of 2-iodothiophene methanol as an etching solution. Given its relatively low polarity, 2-iodothiophene is anticipated to mitigate the corrosion of grain boundaries. The introduction of 2-iodothiophene-treated NC film is anticipated to decrease the interface defects and extend the optical response to longer wavelengths. The concentration of the 2-iodothiophene will evidently exert influence on the back contact resistance and spectrum response of NC solar cells. It was concluded that device performance increases with the increase in 2-iodothiophene concentration from 0 to 5 μg/mL and then degrades when further increasing the 2-iodothiophene concentration (Figure S4a). From the current density–voltage (*J-V*) curve (Figure S4a) and Table S1, it was determined that better device performance is attained by adopting a low 2-iodothiophene concentration, while device performance degrades with the rise in 2-iodothiophene concentration, primarily suffering from low *J*sc and fill factor. Devices treated with ammonium iodide also exhibit a similar trend (Table S2). Figure 3b illustrates the *J-V* characteristics of the champion device for the control device (treated with NH_4_I methanol) and 2-iodothiophene-treated and untreated devices. The corresponding values for short-circuit current density (*J*sc), open-circuit voltage (*V*oc), fill factor (FF), and power conversion efficiency (PCE) are detailed in Table 2.

For reproducibility, four identical devices were fabricated in each group, and the reported values represent the average of the measured data. The error margins reflect the device-to-device variation within each group. Figure 3a shows the schematic structure of the device without any iodized salt treatment. The untreated device exhibited a *V*oc of 0.562 ± 0.03 V and a *J*sc of 9.64 ± 0.02 mA/cm^2^. In contrast, the NH_4_I-treated device showed a *V*oc of 0.490 ± 0.03 V, a *J*sc of 12.97 ± 0.02 mA/cm^2^, and an FF of 47.49 ± 0.01%, resulting in a PCE of 3.08 ± 0.02%. The device treated with 2-iodothiophene displayed an improved *V*oc of 0.598 ± 0.03 V, a *J*sc of 12.35 ± 0.02 mA/cm^2^, and an FF of 52.18 ± 0.01%, leading to a higher PCE of 3.85 ± 0.02%. Consequently, the 2-iodothiophene-treated devices achieved 25% higher efficiency than the NH_4_I-treated control devices. It should be pointed out that the devices prepared in this way are homogeneous with an efficiency difference of less than 10% in each single film (for each type, over 20 devices are made from 4 individual films). To further eliminate the interface defects between CdTe NC alloy active layers and Au back contact, an optimized annealing strategy should be adopted in NC solar cells. From another perspective, the existing high contact resistance will likely accelerate the carriers’ recombination and degrade device performance. To further improve device performance, the ITO/ZnO/CdSe/CdTe/2-iodothiophene (5 μg/mL) substrates were heat-treated at different temperatures. According to our previous work [33], higher temperatures lead to poorer ITO conductivity; therefore, the annealing temperature was controlled below 400 °C in this study. As shown in Figure 2b and Table 3, the 250 °C-treated device was processed to display the following figures of merit: *J*sc of 12.79 mA/cm^2^, *V*oc of 0.628 V, FF of 53.4%, and high PCE of 4.31%, which is significantly higher than the controlled device (3.08%). Lower or higher annealing temperature degraded device performance.

**Figure 4:**
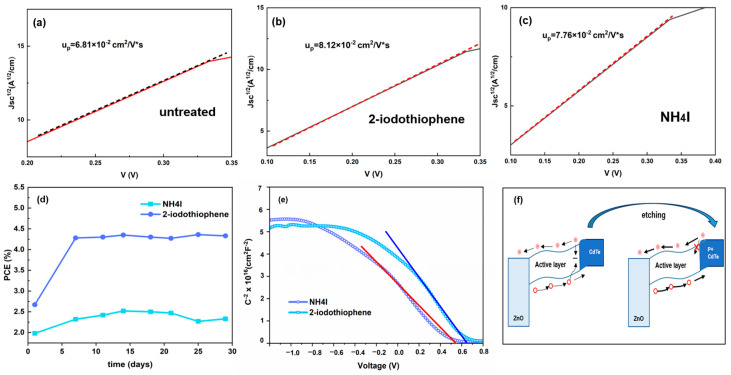
The hole mobility of the CdTe NC thin films: (**a**) untreated, (**b**) 2-iodothiophene-treated, and (**c**) NH_4_I-treated; (**d**) stability of the NH_4_I and 2-iodothiophene-treated devices; (**e**) *C*^−2^-*V* characteristics of the NH_4_I and 2-iodothiophene-treated devices; (**f**) schematic illustration of the carrier transport mechanism at the CdTe interface.

The EQE curves of NC devices (Figure 3d) denote a prominent response confined to the entire range of 400–900 nm; when they are integrated, current densities of 15.86 mA/cm^2^ and 17.93 mA/cm^2^ are predicted, respectively, which is consistent with our AM 1.5 G measurements (Figure 3b). The device subjected to 2-iodothiophene treatment exhibited a dark current density that was an order of magnitude lower than that of the control device (refer to Figure 3e), indicating that leakage current at the anode can be effectively curtailed. It is noteworthy that the *R*s of the 2-iodothiophene-treated device is lower than that of the control devices. We speculated that at an optimized annealing temperature, more CdO will be removed and more Te element will be generated, which increases the hole concentration and results in a decrease in *R*s, and a higher FF and *J*sc are expected in this case. It is worth pointing out that the device is very stable when kept under room light conditions, with less than 5% degradation found over 30 days (Figure 4d). We believe that by further optimizing the device fabrication process/device structure, such as using more optimized active layer thickness (~400 nm), carefully controlling the annealing strategy, or using a more suitable back contact, device performance with PCE up to 10% will be obtained.

In order to determine the charge transport characteristics of CdTe NCs treated with 2-iodothiophene, hole-only devices were constructed with the following layered structure: ITO/CdTe (160 nm)/Au (70 nm). The carrier mobility values were obtained in accordance with the subsequent equation [34]:

$$J=\frac{9}{8}\frac{\varepsilon_{0}\varepsilon_{r}\mu_{p}{\left(V-V_{bi}-V_{s}\right)}^{2}}{L^{3}}$$

where *ε*_0_ represents the permittivity of free space, *ε_r_* is the relative dielectric constant of CdTe, *L* denotes the thickness of CdTe NCs, *μₚ* signifies the hole mobility, *V* indicates the applied voltage, *V_s_* signifies the voltage drop, and *V_bi_* corresponds to the built-in voltage. As exhibited in Figure 4a–c, the mobilities of the untreated, 2-iodothiophene-treated, and NH_4_I-treated CdTe NC thin films are 6.81 × 10^−2^, 8.12 × 10^−2^, and 7.76 × 10^−2^ respectively. The value of the 2-iodothiophene-treated NCs markedly surpasses that of the untreated and the NH_4_I-treated ones. It is posited that the formation of elemental Te at the interface augments the hole concentration, thereby resulting in enhanced carrier mobility.

To further investigate the effect of 2-iodothiophene on the built-in electric field of NC solar cells, capacitance-voltage analysis was carried out with the increase in bias voltage at a constant frequency of 1000 Hz. As shown in Figure 4a, *C*^−2^-*V* curves were plotted based on the Mott–Schottky equation [35]:

$$C^{-2}=\frac{2(V_{bi}-V)}{{A^{2}q\varepsilon }_{0}\varepsilon_{r}N_{A}}$$

where *A* is the device active area, *ε* is the relative dielectric constant (10.6), *ε*_0_ is the permittivity of vacuum, *V_bi_* represents the built-in potential, and *N_A_* is the net acceptor concentration. The *V_bi_* is extracted at a forward bias from the slope and the extrapolated intersection with the x axis. A higher *V_bi_* (0.628 V) of the device with 2-iodothiophene than that (0.549 V) of the control device was observed, which agrees well with their *V*_oc_ values from the *J-V* curves. Figure 4f illustrates a schematic representation of the energy alignment in the NC device without 2-iodothiophene treatment. It was observed that treatment with 2-iodothiophene facilitates the formation of a p^+^-doped interface, thereby reducing carrier recombination at the interface. Consequently, the energy barrier for hole transfer from CdTe to Au is diminished, enhancing carrier collection efficiency in this instance.

## 4. Conclusions

In conclusion, the successful fabrication of CdTe NC solar cells with 2-iodothiophene treatment has been achieved by meticulously controlling the concentration of the 2-iodothiophene methanol solution. The formation of a Te-rich layer subsequent to 2-iodothiophene treatment leads to diminished carrier recombination at the interface and reduced contact resistance. CdTe NC thin-film devices subjected to 2-iodothiophene treatment exhibit enhanced power conversion efficiencies (PCEs) of up to 4.31%, surpassing those of untreated and NH_4_I-treated devices. The superior performance of these devices is predominantly attributed to the reduced recombination losses at the CdTe/Au back electrode interface, as substantiated by an array of electrical and optical assessments. Additionally, devices treated with 2-iodothiophene demonstrate exceptional operational stability, exhibiting no degradation under ambient conditions. This study underscores the efficacy of this straightforward 2-iodothiophene treatment method, which holds promise for integration into future manufacturing systems for low-cost, solution-processed CdTe NC solar cells.

## Figures and Tables

**Table 1:** Summarized XPS peaks of Cd3d, I3d, O1s, and Te3d elements for CdTe NC films with/without 2-iodothiophene treatment.

Condition	Cd3d5/2	Cd3d3/2	Te3d5/2	Te3d3/2	Te/Cd (%)	I3d (%)	O1d (%)
Untreated	404.29	411.09	571.6	582	10.60/16.78	——	32.03
2-iodothiophene	404.55	411.28	571.91	582.41	11.39/16.58	3.99	31.87

**Table 2:** Summarized performance of devices with/without iodized salt treatment.

Condition	*V*oc (V)	*J*sc (mA/cm^2^)	FF (%)	PCE (%)	*R*s (Ω*cm^2^)	*R*Sh (Ω*cm^2^)
untreated	0.562	9.64	45.62	2.48	98.45	1433.64
NH_4_I	0.49	12.97	47.49	3.08	99.48	1424.65
2-iodothiophene	0.598	12.35	52.18	3.85	95.83	2083.33

**Table 3:** Summarized device performance under different annealing temperatures.

Temperature (°C)	*V*oc (V)	*J*sc (mA/cm^2^)	FF (%)	PCE (%)	*R*s (Ω*cm^2^)	*R*Sh (Ω*cm^2^)
150	0.556	8.55	50.25	2.39	97.48	1423.65
200	0.454	14.14	43.43	2.78	70.79	810.59
250	0.628	12.79	53.4	4.31	81.19	1397.13
300	0.442	14.86	44.46	2.95	66.78	798.03

## Data Availability

The authors confirm that the data supporting the findings of this study are available within the article and its ESI.

## Supplementary Materials

The following supporting information can be downloaded at: https://www.mdpi.com/article/10.3390/nano15151180/s1, Figure S1: (a) Transmittance spectrum of a 4-layer CdTe thin films with a total thickness of 300 nm; (b) Photograph of the corresponding device; Figure S2: AFM images of CdTe NCs thin films with 2-iodothiophene etching for (a) control; (b) 10ug/mL; (c) 15 ug/mL and (d) 60 ug/mL; Figure S3: AFM images of CdTe NCs thin films with NH4I etching for (a) control; (b) 500 ug/mL; (c) 1 mg/mL; Figure S4: J-V characteristics of CdTe/CdSe NCs solar cells (a) with different 2-iodothiophene concentration etching and (b) with different NH_4_I concentration etching; Table S1: Summarized device performance with different 2-iodothiophene concentrations under 150 ℃ treated; Table S2: Summarized device performance with different NH_4_I concentrations.
