# Supplementary material for: Iodide Salt Surface Etching Reduces Energy Loss in CdTe Nanocrystal Solar Cells
Source: Nanomaterials (Basel). 2025 Jul 31;15(15):1180. doi: 10.3390/nano15151180 (PMC12348779; doi:10.3390/nano15151180)
Supplement: Supplementary file 1 [file nanomaterials-15-01180-s001.zip › nanomaterials-3751176-supplementary.pdf]

Iodide salt surface etching reduces Energy Loss in CdTe nanocrystals Solar Cells

Jielin Huang [1], Xuyang Wang [1], Yilin Chen [1], Zhenyu Chen [1], Qiaochu Lin [1], Qichuan Huang [1], Donghuan Qin [1,2,*]

† These authors contributed equally to this work.

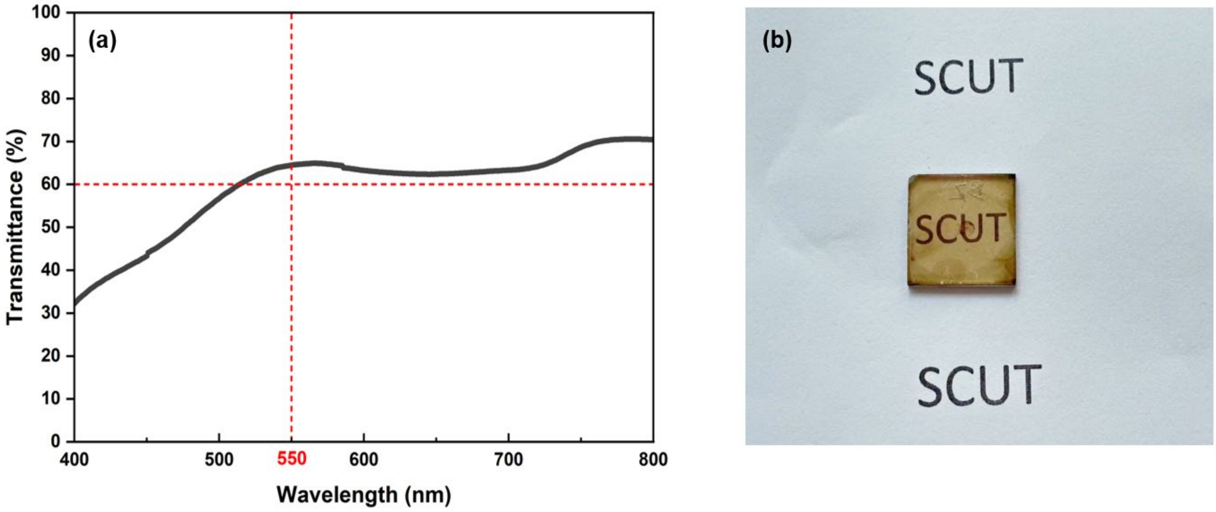

Figure S1 (a) Transmittance spectrum of a 4-layer CdTe thin films with a total thickness of 300 nm; (b) Photograph of the corresponding device.

[1] School of Materials Science and Engineering, South China University of Technology, Guangzhou 510640, China; 202230271209@mail.scut.edu.cn (J.H.); 202230274026@mail.scut.edu.cn (X.W.); msylchen@mail.scut.edu.cn (Y.C.); 202230270257@mail.scut.edu.cn (Z.C.); 202130273624@mail.scut.edu.cn (Q.L.); hqcscut@outlook.com (Q.H.)

[2] State Key Laboratory of Luminescent Materials & Devices, Institute of Polymer Optoelectronic Materials & Devices, South China University of Technology, Guangzhou 510640, China;

* Correspondence: qindh@scut.edu.cn (D.Q.); Tel.: +86-020-8711-4346 (D.Q.);

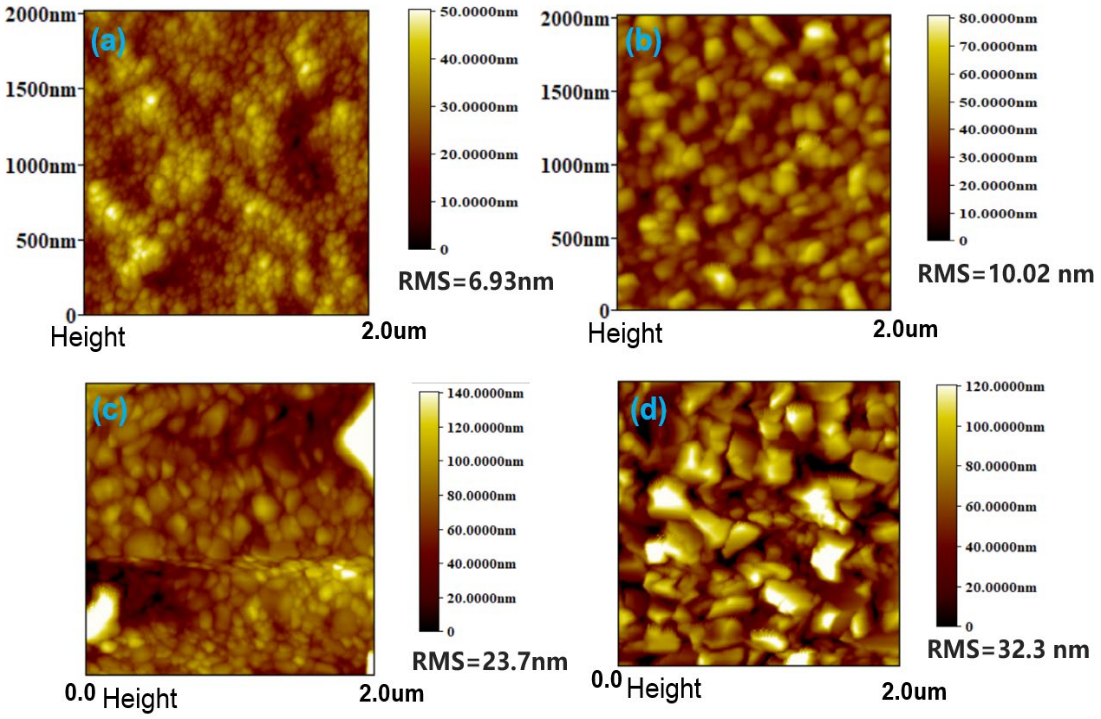

Figure S2 AFM images of CdTe NCs thin films with 2-iodothiophene etching for (a) control, (b) 10 ug/mL (c) 15 ug/mL and (d)60 ug/mL.

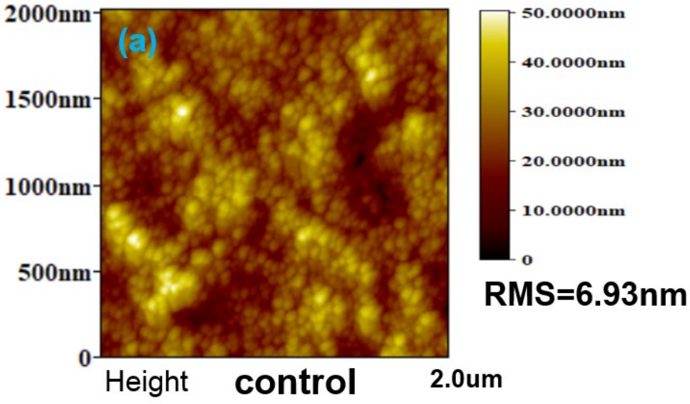

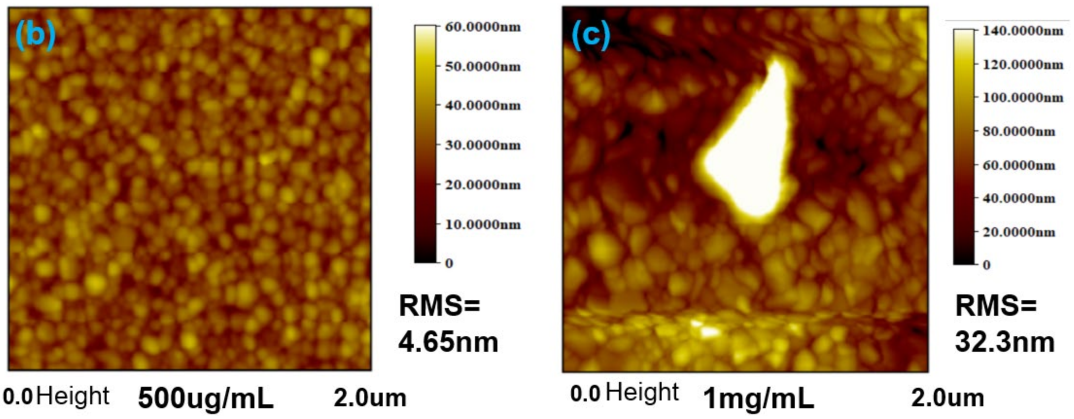

Figure S3 AFM images of CdTe NCs thin films with NH_4I etching for (a) control, (b) 500 ug/mL (c) 1 mg/mL.

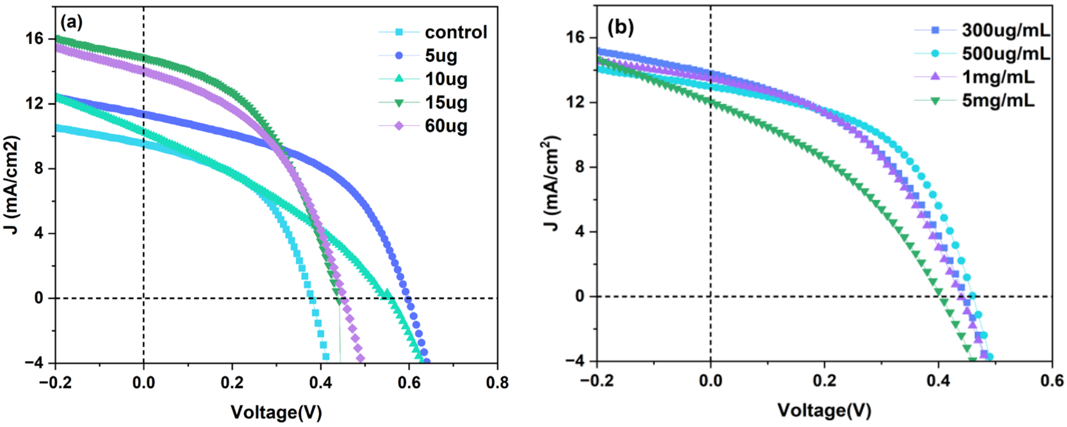

Figure S4 *J-V* characteristics of CdTe/CdSe NCs solar cells (a) with different 2-iodothiophene concentration etching and (b) with different NH_4I concentration etching.

Table S1 Summarized device performance with different 2-iodothiophene concentrations under 150 °C treated.

Condition	V_{oc} (V)	J_{sc} (mA/cm^2)	FF (%)	PCE (%)
5 ug/mL	0.598	11.38	48.19	3.29
10 ug/mL	0.544	10.31	32.99	1.86
15 ug/mL	0.442	14.87	44.48	2.95
60 ug/mL	0.454	14.14	43.43	2.79

Table S2 Summarized device performance with different NH_4I concentrations.

**Condition**	V_{oc} **(V)**	J_{sc} **(mA/cm^2)**	**FF (%)**	**PCE (%)**
300 ug/mL	0.45	13.79	42.73	2.65
500 ug/mL	0.46	12.98	50.46	3.11
1 mg/mL	0.44	13.44	44.13	2.67
5 mg/mL	0.45	12.03	36.28	1.79
